# How Does Opium Dependency Affect the Myocardial Infarction Outcomes? -

**DOI:** 10.31661/gmj.vi.3545

**Published:** 2025-05-18

**Authors:** Shahriar Shirkhoda, Somayyeh Norouzi, Mansoor Namazi, Seyed Amir Hoseini

**Affiliations:** ^1^ Department of Cardiology, Shahid Beheshti Hospital, Qom University of Medical Sciences, Qom, Iran; ^2^ Cardiovascular Research Center , Rajaie Cardiovascular Institute , School of Medicine , Iran University of Medical Sciences , Tehran , Iran

**Keywords:** Opioid, Acute Coronary Syndrome, Coronary Artery Diseases, Arrhythmias, Coronary Stenosis

## Abstract

**Background:**

Acute Coronary Syndrome (ACS) remains a leading global cause of mortality, with multiple established risk factors. While smoking is widely recognized, opium use poses a significant health concern, particularly in developing nations. Despite traditional beliefs suggesting potential cardioprotective effects, growing evidence indicates a detrimental impact of opium consumption on cardiovascular health. This study aimed to investigate the association between opium dependence and the clinical outcomes of patients experiencing ACS.

**Materials and Method:**

This study, conducted on 165 patients with ST-elevation myocardial infarction (STEMI) and Non-ST-elevation myocardial infarction (NSTEMI) referred to the Qom Heart Center, Qom, Iran from 2022 to 2024. The patients were categorized in two groups: 81 opium-dependent patients based on DSM-V criteria as a case group and 84 non-opium-dependent patients as a control group, and their clinical outcomes were compared.

**Results:**

Coronary artery involvement was significantly more severe in opium-dependent patients ie three vessel disease (3VD) or left main stenosis (LM) involvement in the opium-dependent group was 48.1% versus 28.6% in the non-opium-dependent group, (P-value=0.01). Prevalence of atrial fibrillation (AF) and delay in hospital admission and the hospitalization days were higher in the opium-dependent group. LVEF at the admission did not differ between the two groups, but left ventricle ejection fraction (LVEF) three months later was higher in the opium-dependent group. Rehospitalization, arrhythmia, mechanical complications, need for CABG, and mortality during the initial hospitalization did not differ between the two groups. The mortality of the patients in the six-month follow-up was 6.5% in the opium-dependent group and 8.6% in the non-opium-dependent group (P-value=0.61) which did not show a significant difference. This issue shows the lack of significant role of opium consumption in the mortality of patients.

**Conclusion:**

Our findings suggest a strong association between opium dependence and a more severe clinical presentation of ACS, characterized by a higher burden of coronary artery disease. Although initial LVEF recovery appeared faster in opium-dependent individuals, the overall impact on long-term mortality remained inconclusive within the six-month follow-up period. These findings underscore the critical need for further research to elucidate the complex interplay between opium dependence and cardiovascular outcomes in ACS patients, including long-term follow-up and exploration of potential underlying mechanisms.

## Introduction

Cardiovascular disease (CVD) remains a leading global health challenge, characterized by a complex interplay of risk factors that contribute to its development and progression [1].

While traditional risk factors such as hypertension, diabetes, and dyslipidemia are well-established, a substantial portion of the disease burden remains unexplained. Emerging research has focused on the role of inflammation, genetic predisposition, and lifestyle factors in CVD pathogenesis [2][3].

There are other risk factors, including opium consumption, which its relationship with the development of heart diseases has not yet been conclusively proven [4].There is a traditional belief among Asians that opium consumption can reduce the incidence of cardiovascular diseases [5]. In many developing countries, opium abuse is the second health challenge after smoking [5]. This issue seems to originate from the same traditional belief that people believe that the use of opiates causes protective effects on metabolic and cardiovascular diseases. The important point is that this belief exists more among elderly people who are more at risk of cardiovascular disease or have other risk factors [6].

It has been observed that opioids have many effects on different body systems by acting on delta, kappa and gamma receptors [7]. On the other hand, it seems that the protection of the heart against ischemia depends on two receptors, delta and kappa [8]. Opium is a substance that significantly affects the cardiovascular system, and usually patients with coronary artery diseases tend to it [9]. Although some studies have shown that opium use can moderate some risk factors for coronary artery disease, these findings have been contradicted in many other studies, and other studies show that opium use is harmful [10][11]. Studies till now indicate no significant difference in mortality rates between opium-dependent and non-opium-dependent myocardial infarction (MI) patients [12][13]. However, some studies suggest a potential trend towards higher mortality in opium users, though not statistically significant [13] Opium-dependent patients have a significantly higher rate of re-hospitalization due to heart problems compared to non-opium-dependent patients [13] The duration of hospital stays is longer for opium-dependent patients [14] Opium dependency does not significantly affect cardiovascular risk factors such as ejection fraction or post-AMI morbidity [9] Opium consumption is associated with a higher risk of long-term adverse cardiovascular outcomes, including all-cause mortality and major adverse cardiac and cerebrovascular events (MACCE) [15] Opium can induce metabolic changes that may negatively impact the cardiovascular system, such as increased inflammation and oxidative stress [16] .

Although there is evidence for a protective and/or non-protective role of opiate use in patients with coronary artery disease, the exact role of opiate is still in question thus, the effect of opium consumption on the incidence and its role in the prognosis of people with coronary artery diseases and its risks are still unknown. [7][17]. Also, the role of opium consumption in the prognosis of users is not clearly defined. Therefore, in the following study, we decided to evaluate the role of opium consumption on the clinical outcome of patients with coronary artery diseases.

## Materials and Methods

This study was carried out in a descriptive and analytical manner on patients with ST‑elevation myocardial infarction (STEMI) and Non-ST‑elevation myocardial infarction (NSTEMI) which categorized as opium-dependent group (case group) and non-opium-dependent group (control group) .This clinical study as a retrospective study (based on patient history) and prospective (patient follow-up) study six months after coronary revascularization approved by the research council of Qom University of Medical Sciences and the university ethics committee. In order to participate in the study from all patients written informed consent was obtained. Patients with coronary artery disease hospitalized at the Shahid Beheshti Hospital in Qom, Iran were included in the study.

Inclusion criteria: STEMI and NSTEMI patients from 35 to 65 years old.

Exclusion criteria: unstable vital signs of patients, non-cooperation of patients in conducting the study, patients with diabetes, previous history of acute coronary syndrome with PCI or Coronary artery bypass surgery (CABG) were excluded from the study.

Patients of the case group were those STEMI and NSTEMI patients who were considered opium-dependent based on DSM-V criteria [18]. Patients of the control group were STEMI and NSTEMI patients who never had history of opium use. It should also be noted that in order to eliminate the possibility of the influence of other risk factors of coronary artery diseases on the study results, patients of both groups who had other risk factors such as diabetes and previous history of acute coronary syndrome were excluded from the study. The patient’s data based on their history of opium consumptions, and history of other risk factors, coronary artery angiography results, and echocardiography findings was prepared. Clinical condition of all patients during six months after coronary intervention was assessed by echocardiography and physical exam. The main outcomes of this study were summarized as in-hospital mortality, mortality during six-months, left ventricular ejection fraction before and post coronary intervention, the number of involved coronary vessels, and the number of cases referred for open heart surgery.

### Ethics Approval and Consent to Participate

The study complies with the Declaration of Helsinki. Ethical approval was obtained from the Ethics Committees of Qom Cardiovascular Medical and Research Center, Qom University of Medical Sciences, Qom, Iran IR.MUQ.REC.1402.221 Written informed consent was obtained from the participants.

## Statistical Analysis

All recorded data, including, the number of coronary artery stenosis, age, gender, and etc., were analyzed by Statistical Package of Social Science version (SPSS) software version 26 (SPSS, Inc., Chicago, IL, USA). Continuous variables according to the type of distribution were expressed as mean ± standard deviation (SD). Continuous values were compared by independent t-test or Mann-Whitney U. Categorical data were analyzed using chi-square test or Fisher’s exact test. Regression tests were used to check the relationship. A two-sided P<0.05 was considered statistically significant.

## Results

**Table:** Table1. Baseline demographic and clinical characteristics of the study population according to the opium dependency

		Opium dependent N=81 (%)	Non-Opium dependent N=84 (%)	P-value
Sex	Male	70(86.4%)	55(65.5%)	0.002
	Female	11(13.6%)	29(35.5%)	
Age(Years)	Mean ±SD	59.25±11.74	57.10±13.28	0.273
GFR(ml/min)	Mean ±SD	69.51±17.34	74.05±18.3	0.1
Total Cholesterol(mg/dl)	Mean ±SD	239.3±79.3	232.26±87.27	0.58
LDL Cholesterol (mg/dl)	Mean ±SD	129.6±34.43	126.5±36.28	0.571
HDL Cholesterol(mg/dl)	Mean ±SD	24.81±8.5	32.67±10.2	0.001
Triglyceride(mg/dl)	Mean ±SD	265.2±73.1	259.8±77.7	0.64
Hb(g/dl)	Mean ±SD	11.3±2.9	13.1±3.4	0.001
Delay in hospital referral (Hours)	Mean ±SD	6.07±4.55	4.27±3.59	0.001
Hospitalization days	Mean ±SD	8.18±2.26	6.28±2.35	0.001
Pre-DM state(Yes)	Hb A1-C: 5.7-6.4%	7(8.6%)	6(7.1%)	0.721
HTN(Yes)	BP ˃140/90 mm Hg	19(23.5%)	15(17.9%)	0.374
Smoking(Yes)		72(88.9%)	34(40.5%)	0.001

**GFR:** glomerular filtration rate , **LDL:** low-density lipoproteins, **HDL:** High-density Lipoprotein, **Hb:** Hemoglobin, **Pre-DM:** Pre-Diabetes Mellitus, **HTN:** Hypertension

**Table:** Table2. ACS type, Arrhythmia and angiography result of the patients according to their opium dependency

		Opium dependent N=81 (%)	Non-Opium dependent N=84 (%)	P-value
ACS type	STEMI	57(70.4%)	61(72.6%)	0.749
	Non-STEMI	24(29.6%)	23(27.4%)	
	AF	9(11.1%)	2(2.4%)	0.02
	No Arrhythmia	23(28.4%)	32(38.1%)	
	PVC or non sustained VT	21(25.9%)	22(26.2%)	
Arrhythmia	PAC	16(19.8%)	15(17.9%)	
	AIVR or sustained VT	5(6.2%)	4(4.8%)	0.481
	VF	3(3.7%)	4(4.8%)	
	Asystole	1(1.2%)	2(2.4%)	
	CHB	3(3.7%)	3(3.6%)	
	Normal	5(6.2%)	3(3.6%)	
Angiography Result	SVD	15(18.5%)	33(39.3%)	0.013
	2VD	22(27.2%)	24(28.6%)	
	3VD or LM	39(48.1%)	24(28.6%)	
CABG	Yes	16(19.8%)	11(13.1%)	0.248
	No	65(80.2%)	73(86.9%)	

**ACS:** Acute Coronary Syndrome, **AIVR:** Accelerated idioventricular rhythm, **AF:** Atrial Fibrillation, **CHB:** Complete Heart block, **CABG:** Coronary artery bypass grafting, **STEMI:** ST-elevation myocardial infarction, **PVC:** Premature Ventricular Complex, **VT:** Ventricular tachycardia, **VF:** Ventricular fibrillation, **SVD:** Single Vessel Disease,**2VD:** Two Vessel Disease, **3VD:** Three Vessel Disease, **LM:** Left main coronary.

**Table:** Table3. Outcome of patient in two groups According to their opium dependency

	Opium dependent N=81 (%)	Non-Opium dependent N=84 (%)	P-value
LVEF at admission (%)	35.18±10.53	32.26±9.73	0.06
LVEF after 3 months (%)	45.3±9.12	41.13±9.88	0.005
Death at hospital	4(4.9%)	3(3.6%)	0.66
6- months death	5(6.5%)	7(8.6%)	0.61
readmission	26(36.1%)	30(40.5%)	0.582
Stent-Thrombosis	2(3.3%)	1(1.4%)	0.471
Cardiac Mechanical complication	0	1(1.2%)	0.377

**LVEF:** Left ventricle ejection fraction

Of the 179 patients who were enrolled in this study, 89 patients were in the group of opium users, eight patients of this group were excluded from the study due to non-cooperation, and the remaining 81 patients were studied until the end of the study. In the control group, 90 patients with no history of opium addiction, were initially included in the study, of which six patients did not cooperate and were excluded, and the remaining 84 patients in this group were studied as a control group. Out of the total of 165 patients examined in this study, 125 patients (75.8%) were male and 40 patients (24.2%) were female. The average age of the examined patients in this study was 57.01 ± 12.6 years. The youngest patient in this study was 33-year-old, and the oldest was 65-years old. As outlined in the table-1, the patients of the two groups had a significant difference in terms of gender, which is probably due to the Iranian culture and the greater consumption of opium in Iranian men. Patients in the opium-dependent group had a longer delay before coming to the hospital, and they needed a longer period of hospitalization at the time of hospitalization. There was no significant difference in laboratory tests like glomerular filtration rate (GFR), serum Triglyceride (TG), total Cholesterol, and Low-density lipoproteins (LDL) levels between the two groups. However, High-density lipoprotein (HDL) and serum Hemoglobin (Hb) levels were significantly lower in the opium dependent group. There was no significant difference between the mean age of patients, hypertension (blood pressure more than 140/90 mm Hg), and the number of pre-diabetic patients (Hb A1-C 5.7-6.4%) in the two groups (Table-1).

The patients of the two groups did not differ significantly based on the type of acute coronary syndrome (STEMI, NSTEMI) and types of arrhythmias, but AF arrhythmia was significantly more common in the opium-dependent group in compare to the control group (P-value=0.02). About 48% of opium-dependent patients and 28% of non-opium-dependent patients had stenosis in all three epicardial coronary arteries or left main coronary artery (LM) during coronary angiography. Based on statistical analysis, there was a significant difference between the two groups of patients with coronary artery involvement. However, there was no significant difference between the two groups of patients in terms of the need to perform Coronary artery bypass grafting (CABG) (Table-2).

Echocardiographic findings of the patients demonstrated that the left ventricle ejection fraction (LVEF) of the opioid user group in compare with control group at the beginning of acute coronary syndrome was different, but it was not statistically significant (P-value=0.06). During 3 months fallow up after acute coronary syndrome, LVEF among the patients in the opium-dependent group was higher than non-opium dependent patients and it was statistically significant (P-value=0.005). The mortality rate of the patients had no relationship with their opium consumption (P-value=0.66). The incidence rate of stent thrombosis, readmission due to cardiac events and cardiac mechanical complications were not significantly different between the two groups (P-value˃0.05) (Table-3).

## Discussion

Despite conducting numerous studies on the role of opium consumption in the prognosis of patients with coronary artery diseases, the exact role of this substance in the prognosis and complications of this disease is still in question and is not clearly defined. In the present study, the effect of opium consumption on the clinical outcome of patients with coronary artery diseases was evaluated. In this study, 81 patients in the case group (opium-dependent) and 84 patients in the control group (non-opium-dependent) were enrolled. It was observed that in terms of the severity of coronary artery involvement, the patients of the two groups were significantly different from each other, and it seems that the opium-dependent patients had more involved vessels (3VD or LM involvement in the opium-dependent group was 48.1%, versus 28.6% in the non-opium dependent group (P-value=0.01). This difference could be due to more chronic and longer involvement of coronary arteries in opium consuming patients. Also, the higher prevalence of smoking in the opium-dependent group can be a confounding factor which have hidden role in this difference.

It was also observed that opium dependence had no effect on the frequency of arrhythmias after ACS (P-value=0.481), and the type of acute coronary syndrome (STEMI or non-STEMI) among the patients (the frequency of STEMI was 70.4% in the opium-dependent group versus 72.6% in the non-opium-dependent group, P-value=0.749).

The comparison of the left ventricular systolic function between the two groups demonstrated that at the first day of ACS occurrences, opium-dependent patients had a better left ventricular function, but this difference was not statistically significant, but after three months, the difference between the two groups was significant. The improvement of left ventricular systolic function in opium dependent patients was significantly better than the control group (10.12% improvement in the case group compared to 8.87% improvement in the control group, P-value=0.005). This significant difference in the recovery of left ventricular systolic function could be due to the formation of more collateral vessels in the setting of hypoxia and anemia in the opioid-dependent group in other words opium addiction will lead to a higher prevalence of chronic anemia and chronic hypoxia in the patients so cardiac collateral vessels as a defensive mechanism in the preconditioning state will be developed and these vessels in ACS situation will help the patient to preserve its LVEF more than non-opioid dependent patients.

Delay in going to the hospital among the opium dependent group was about 6.07 hours after the onset of the first heart symptom, but the average delay in the control group was 4.27 hours, which had a significant difference, (P-value=0.001). This delay in going to the medical center in opium dependent group is probably due to the role of opium in controlling the pain of patients since their feeling of chest pain is impaired. Delay in going to the hospital, leads to delay in onset of treatment in this group, so it can be another confusing factor in the clinical outcome of these patients. This means that earlier referral and earlier treatment may be associated with better clinical results in control group versus opium dependent group.

The duration of hospital stay was significantly longer in opium consuming patients than in the non-consuming group (about 8.18 days for the case group compared to 6.28 days for the control group with P-value=0.001). The difference in the length of hospitalization between these patients is probably coincident with more underlying diseases in the opium user group, including anemia and lung diseases. In order to investigate this difference, the patients of the two groups were examined to find out any obstructive pulmonary diseases. In the results, it was observed that 56 patients from the subject group had a history of suffering from obstructive pulmonary diseases, while this disease was reported in only 25 patients in the control group.

Investigating the cause of anemia was beyond the goals of this study and was only seen as a random finding especially in opium dependent group.

The patients of these two groups were not significantly different in terms of the need for re-hospitalization (P-value=0.582), stent thrombosis (P-value=0.471) and occurrence of mechanical complications (P-value=0.377). This shows that opium consumption probably did not play a role in re-hospitalization, stent thrombosis and occurrence of mechanical complications in patients.

Hospital mortality rate was 4.9% in the opium-dependent group and 3.6% in the non-opium-dependent group. It was also observed in the 6-month follow-up that 6.5% of the remaining 77 patients in the opium-dependent group and 8.6% of the remaining 81 patients in the non-opium-dependent group died. The statistical analysis conducted in this section also did not show a significant difference in in-hospital mortality (P-value=0.66) and 6-month mortality (P-value=0.61). This issue shows the lack of significant role of opium consumption in the mortality of patients.

In a study conducted by Gholamreza Davoodi and also stated that the length of hospital stay was significantly longer in opium-dependent patients (11.3 days vs. 8.7 days) [14]. This finding is completely consistent with the results of our study.

Also, Davoodi et al. stated that opium consumption had no effect on left ventricular ejection fraction, hospital mortality, need for re-hospitalization, 6-month mortality and need for revascularization [14]. As can be seen, these results are not consistent with the results of our study in terms of the systolic function of the left ventricle. This inconsistency is probably due to the fact that the left ventricular systolic function in the study of Davoodi et al. was measured only at the beginning of hospitalization, while in our study the results were different at the end of 3 months. However, in our study, the left ventricular function in the opium-dependent group was slightly better than the non-opium-dependent group (44% vs. 42%).

The study by Mohammadali Bayani et al. stated that opium consumption has no protection against dyslipidemia and diabetes [19]. In our study, it was also observed that opium consumption had no protective role against dyslipidemia, and even in HDL examination, it was observed that its level was worse in the opium-dependent group than in the non-dependent group.

In another study conducted in this field, Hamid Reza Javadi et al. stated that opium addiction did not play a large role in hospital mortality [20]. In the results of our study, it was also observed that opium consumption had no effect on hospital and 6-month mortality of patients.

Mohammad Reza Habibi et al reported that the average amount of bleeding after surgery in opium-dependent patients was significantly higher than non-opium-dependent patients [21]. Also, the average duration of dependence on the ventilator, the duration of hospitalization and the incidence of atrial fibrillation were significantly higher in opium-dependent patients [21]. These findings were less noticed in our study, but in our study, it was observed that these patients did not differ from each other in terms of the need for CABG or the occurrence of arrhythmias. This difference could be due to the surgery itself, which was not evaluated in the objectives of our study. It was also observed in our study that the hospitalization rate after acute coronary syndrome was higher in opium-dependent patients than in the control group, which is consistent with the results of Habibi’s study.

Our findings demonstrate a significant association between opium dependence and more extensive coronary artery disease, suggesting a potential link between chronic opium use and accelerated atherosclerosis. The higher prevalence of smoking in the opium-dependent group is a crucial confounding factor, as smoking is a well-established independent risk factor for cardiovascular disease. However, even after considering this, the observed difference in coronary artery involvement suggests that opium use may have a detrimental impact on the cardiovascular system. This could be attributed to various mechanisms, including the potential for opium to induce endothelial dysfunction, promote oxidative stress, and contribute to dyslipidemia and insulin resistance. Further research is warranted to elucidate the specific pathways through which opium may contribute to the progression of coronary artery disease.

While opium dependence did not significantly impact the frequency of arrhythmias, the type of acute coronary syndrome, or in-hospital mortality, it was associated with a delayed presentation to the hospital. This delay in seeking medical attention likely contributes to worse outcomes, as timely intervention is crucial for optimizing treatment and minimizing myocardial damage. The observed improvement in left ventricular systolic function over three months in the opium-dependent group is an intriguing finding. This could be attributed to the development of collateral circulation in response to chronic hypoxia and anemia, which are common complications of long-term opioid use. However, this finding requires further investigation and confirmation in larger studies.

### limitations

This study, being observational in nature, cannot definitively establish a cause-and-effect relationship between opium dependence and the observed cardiovascular outcomes. The relatively small sample size and potential for selection bias may have limited the study’s ability to detect significant differences and accurately represent the broader population of patients with coronary artery disease. Additionally, the presence of unmeasured confounders, such as socioeconomic factors and lifestyle habits, could have influenced the results, as could the limited follow-up period, which may not adequately capture long-term effects. Furthermore, the lack of data on the specific dose and duration of opium use hinders a more nuanced understanding of the impact of different levels of exposure.

The study also acknowledges the potential confounding effects of factors such as smoking, delayed hospital presentation, and the presence of other comorbidities, including chronic obstructive pulmonary disease and anemia. The impact of these factors could not be fully accounted for in the analysis, potentially influencing the observed outcomes. Finally, the findings may not be generalizable to other populations with different demographic characteristics or patterns of opium use.

## Conclusion

Our findings indicate that opium dependence does not improve survival rates in patients with acute coronary syndrome. In fact, these patients tend to have more severe coronary artery disease, complicating revascularization procedures. Additionally, opium-dependent individuals often experience delayed hospital presentation, hindering timely treatment initiation. However, a surprising observation was improved cardiac systolic function in these patients, possibly attributed to the development of collateral blood vessels.

## Conflict of Interest

None.
